# Corporal punishment and violent behavior spectrum: a meta-analytic review

**DOI:** 10.3389/fpsyg.2024.1323784

**Published:** 2024-02-07

**Authors:** Qingna Pan, Siru Chen, Ying Qu

**Affiliations:** ^1^School of Criminal Investigation in People’s Public Security University of China, Beijing, China; ^2^Investigation Department, Hunan Police Academy, Changsha, China; ^3^School of Business, Hunan Institute of Technology, Hengyang, China; ^4^Department of Psychology at Liaoning Normal University, Dalian, China

**Keywords:** corporal punishment, violent behavior spectrum, meta-analysis, children, VBS

## Abstract

Corporal punishment is believed to precede various forms of violent behavior, yet prior research has yielded inconsistent findings, partly due to variations in violent types and other factors. This meta-analysis systematically reviewed 35 studies including 144 effect sizes (comprising a total sample size of 159,213) investigating the association between corporal punishment and a spectrum of violent behaviors called Violent Behavior Spectrum (VBS). Additionally, meta-regressions were conducted to explore the moderating impact of punishment severity, violence type and cultural context. Our findings indicated a significant positive relationship between corporal punishment and VBS (*r* = 0.238, 95%, CI [0.176, 0.300]). Notably, punishment severity was found to influence the strength of this association. Namely, The more severe the corporal punishment, the more likely it is to lead to VBS. These results enhance our understanding of the intricate connection between corporal punishment and various forms of violence, providing valuable insights for both parenting practices and policy development.

## 1 Introduction

The Violent Behavior Spectrum (VBS), referring to the range of violent behavioral manifestations along a continuum of severity, poses critical implications for psychology, criminology, and policymaking (Huesmann, 2018). Elucidating factors underlying VBS is vital for illuminating etiology and informing interventions and policies, particularly regarding the connection between corporal punishment and violence (Durrant and Ensom, 2012). Considerable research has examined potential links between corporal punishment and aggressive behaviors (Gershoff, 2002). However, studies predominantly focused on aggression, with limited exploration of the VBS continuum. Moreover, past studies did not distinguish clearly between aggression and violence, obstructing examination of VBS associations (Larzelere, 1996). Finally, the relationship between corporal punishment and VBS remains ambiguous, with evidence both affirming and refuting associations (Straus, 1997; Larzelere, 2000). These inconsistencies likely stem from variations in punishment severity and violent types or other potential moderators. Therefore, a focused meta-analysis quantitatively synthesizing the literature on corporal punishment and VBS is essential.

### 1.1 Defining and measuring corporal punishment

Definitions of corporal punishment differ across studies. This analysis adopts an inclusive definition of corporal punishment as disciplinary tactics leveraging mild pain to punish misbehaviors (Straus, 1997). Common forms include spanking, slapping, and ear pulling without significant injury. Based on severity, corporal punishment ranges from mild to severe (Larzelere, 2000). The primary distinction between corporal punishment and abuse involves the harm inflicted, with discipline as the former’s aim and anger expression as the latter’s purpose. Consequently, experiencing abuse versus corporal punishment may yield divergent outcomes (Gershoff, 2002). The relationship between abuse and violence has achieved relatively consistent conclusions. However, the association between corporal punishment and various violence forms remains contentious (Larzelere, 2000). Hence, this analysis focuses on the association between corporal punishment and diverse violence types, excluding studies on parental abuse.

Precisely measuring corporal punishment requires established tools like the Corporal Punishment History Scale and the Conflict Tactics Scale (Smith et al., 2015; Lorber and Slep, 2018). The Corporal Punishment History Scale collects individuals’ frequency of receiving parental corporal punishment during growth through questionnaire surveys. The Conflict Tactics Scale asks in detail about various disciplinary strategies used by parents in education to assess the use of corporal punishment. Both scales have good reliability and validity and are the main tools for assessing corporal punishment experience in current research.

### 1.2 Defining and measuring violent behavior spectrum

The Violent Behavior Spectrum (VBS) refers to a comprehensive framework encompassing a continuum of behaviors characterized by varying violence or aggression degrees (Sánchez-Sansegundo et al., 2020). The spectrum ranges from minor acts like verbal disputes and property destruction to severe acts like armed assault and homicide (Brent, 2011). VBS emerged as an integrative model recognizing violence’s multidimensional nature beyond dichotomous classification into aggression presence or absence (Patrick and Drislane, 2015). This conceptualization enables studying factors influencing violence risk across the full severity spectrum. This analysis includes externalizing problems, antisocial behavior, all aggression types, all violence types, and violent crime in the VBS. Although distinct, studying antisocial and violent behavior under the unified VBS framework has merit. First, some antisocial acts involve violence. Second, both can infringe upon others’ rights and well-being. Third, robust violence research provides theoretical models and methodological tools elucidating antisocial behavior mechanisms and management. Fourth, similar developmental and socio-environmental factors may underlie both behaviors’ manifestation (McCord, 1988). Research on the two behaviors can thus advance understanding of individual variations and inform prevention and correctional programs.

The VBS can be assessed through various quantitative and qualitative methods. Self-report tools and behavioral observations document violent acts directly, while official crime and arrest records provide objective severe violence data. Implicit measures like the Weapons Identification Task gage automatic violence associations (Bushman, 2018). Qualitative interviews can elucidate subjective violence perpetration experiences. A multi-method approach combining self-reports, observations, convictions records, and implicit tests enables robust VBS measurement across the continuum (Jacques and Wright, 2008; Sánchez-Sansegundo et al., 2020).

Established instruments assess VBS dimensions. The Buss-Durkee Hostility Inventory measures violence-proneness through subscales like assault and indirect hostility Established instruments assess VBS dimensions. The Buss-Durkee Hostility Inventory measures violence-proneness through subscales like assault and indirect hostility (Buss and Durkee, 1957). The Lifetime Assessment of Violent Acts captures severe violent offenses (Flannery et al., 2007). Governmental criminal records provide objective individual arrest and conviction data (McCord, 1988). Selecting suitable tools and employing them in combination enhances the measurement of VBS (Coccaro et al., 1997).

### 1.3 Theoretical perspectives

Theoretical perspectives offer differing propositions on corporal punishment’s impacts on the VBS. Social learning theory posits corporal punishment can propagate aggressive tendencies by modeling and reinforcing violence, potentially elevating VBS risk (Bandura, 1978). In contrast, control theories propose that non-abusive discipline promotes self-regulation and socialization, suggesting moderate corporal punishment may not increase VBS risk (Gottfredson and Hirschi, 1990). Attachment theories highlight secure caregiver attachment may buffer corporal punishment’s effects on VBS (Sroufe, 2005). Additionally, general aggression models posit frequent corporal punishment use fosters an externalizing cognitive lens toward relationships that feeds into VBS (Huesmann and Kirwil, 2007).

The theory of social moral development posits that morality should not merely be understood as a set of behavioral rules but rather as a mechanism regulating societal relationships. Morality is defined as an interactive process between individuals and social phenomena, shaped by their societal connections (Kohlberg, 1976; Emler, 1987). Reward and punishment constitute fundamental means through which adults intervene when moral norms are being upheld or transgressed (Eriksson et al., 2017; Ziv et al., 2021). Thus, when employed to uphold moral standards within certain parameters, corporal punishment may not necessarily escalate subsequent aggressive behavior.

Understanding this relationship is particularly crucial for child development as children are sensitive to social norms (McAuliffe et al., 2017). Moreover, children’s comprehension of corporal punishment influences its link with aggression. Research illustrates children aged 5–9 prefer withdrawing from unfair individuals over punishing them (Lee and Warneken, 2020). Recent findings reveal 21-month-olds expect bystanders to engage in third-party punishment of antisocial agents (Geraci, 2021; Geraci and Surian, 2021). Conceptualizing punishment of aggression and rewards for prosocial acts as means to maintain order, children likely endorse such measures, thereby mitigating potential adverse outcomes like aggression. Overall, theoretical corporal punishment and VBS links require further empirical investigation.

### 1.4 Previous empirical research

Empirically, considerable research reveals a positive association between corporal punishment and aggressive behavior (Gershoff, 2002). However, some studies indicate moderate corporal punishment does not increase child aggression (Larzelere, 1996). A meta-analysis concluded corporal punishment predicts higher childhood aggression regardless of baseline levels (Gershoff and Grogan-Kaylor, 2016). Evidence indicates a dose–response relationship, with more frequent corporal punishment linked to greater aggression (Taylor et al., 2017). Conclusions are mixed regarding links between corporal punishment and specific violent motives. Moreover, the longitudinal impacts of childhood corporal punishment on adult violent criminality along the spectrum remain underexplored (Afifi et al., 2017). The correlation between corporal punishment and aggression may vary compared to its associations with other violent behaviors like bullying, violence, and violent crime (Zhu et al., 2017). Further research should elucidate the nuances between corporal punishment and the multidimensional VBS construct.

### 1.5 Potential moderators

The associations between corporal punishment and VBS may influenced by potential moderating factors. The strength and intensity of this relationship are susceptible to the nuanced influences of intervening variables, including the severity of punishment (Larzelere, 1996; Gershoff, 2002) and the specific category of behavior within the violent behavior spectrum. Firstly, the severity of punishment emerges as a pivotal factor influencing the association between corporal punishment and the violent behavior spectrum. Distinct levels of corporal punishment may yield divergent impacts on violent behavior. For instance, mild corporal punishment might only contribute to an escalation of violent tendencies, while severe corporal punishment could be more prone to eliciting actual acts of violence. Consequently, considering the intensity and frequency of corporal punishment is imperative in any analysis. Secondly, the specific category within the violent behavior spectrum emerges as yet another possible moderating variable. The violent behavior spectrum encompasses a wide array of behaviors, ranging from less severe acts like verbal disputes to more extreme acts like armed assaults and homicide. The impact of corporal punishment on an individual’s behavior within this spectrum may diverge substantially based on the particular category of violent behavior under consideration.

Other factors that might impose influence on corporal punishment-VBS include developmental stage (Steinberg, 2009), publication year, culture (Lansford et al., 2005), gender, and measurement tools. Given the absence of a comprehensive integrated framework for these factors, this study seeks to investigate them in an exploratory fashion, aiming to comprehend their possible functions in moderating the relationship between corporal punishment and VBS.

### 1.6 Previous meta-analyses and current study

Several influential meta-analyses have examined the relationship between corporal punishment and child outcomes. Gershoff conducted pioneering research differentiating corporal punishment from abuse and aggression from criminal behaviors (Gershoff, 2002; Gershoff and Grogan-Kaylor, 2016). Findings indicated children subjected to corporal punishment exhibit higher aggression and antisocial tendencies, with impacts potentially extending into adulthood. Utilizing updated techniques, replicated and expanded upon this earlier meta-analysis, further substantiating the negative sequelae of corporal punishment (Gershoff and Grogan-Kaylor, 2016). However, Larzelerea and Kuhn discovered in their meta-analysis that the potential negative impacts of corporal punishment on children hinge on the severity of the punishment and contextual factors (Larzelere and Kuhn, 2005). Mild corporal punishment, under certain circumstances, may prove to be an effective disciplinary strategy. Paolucci and Violato collected 70 empirical studies to explore the relationship between corporal punishment and children’s negative affect, behavior problems, and cognition development (Paolucci and Violato, 2004). The research found that corporal punishment had no significant impact on cognition development, only small negative impacts on negative affect (*r* = 0.20) and behavior problems (*r* = 0.21). The study also called for further in-depth research on potential moderating variables. To get closer to the causal relationship, Ferguson’s meta-analysis solely included longitudinal studies and found weaker correlations between corporal punishment and aggression, antisocial behavior, and cognitive deficits (Ferguson, 2013). This meta-analysis indicated harsh punishment poses greater risk, while mild corporal punishment may not correlate with child problems. Ferguson suggested corporal punishment effects are context-dependent and prohibition may be unwarranted (Ferguson, 2013). In summary, these meta-analyses found inconsistent results, which necessitate further meta-analytic exploration due to the inconsistencies.

While informative, contradictions in prior findings reveal gaps regarding the contexts and mechanisms linking corporal punishment to violent outcomes. The present study aims to contribute uniquely to the literature by comparing corporal punishment effects on a spectrum of aggressive behaviors, from bullying to criminality. Additionally, factors moderating this relationship require clarification. By addressing limitations and integrating previous evidence, the current research tries to provide perspectives to inform practices and policies around parenting and violence prevention. Our central hypothesis is that there is a positive correlation between corporal punishment and the violent behavior spectrum.

## 2 Method

The literature search for this meta-analysis was conducted following PRISMA reporting guidelines for the final report (Page et al., 2021). Electronic databases, including PubMed, PsycINFO, Proquest and Web of Science were searched using a combination of keywords and Boolean operators. The search strategy was designed to include studies published between 1950 and 2023, and it focused on the following key terms: (Corporal punishment or physical punishment or spanking or beating or caning or flogging or hitting or smacking or Strict parenting or Coercive parenting or Punitive discipline or Harsh discipline) and (Violent crime or Violent offense or Crime of violence or Violent act or Violent behavior or Aggressive crime or Violent conduct or Violent delinquency or Violent wrongdoing or Violent transgression). In addition to electronic databases, a manual search of relevant journals, conference proceedings, and reference lists of identified studies was performed to ensure completeness (Cooper and Patall, 2009).

Studies were considered eligible for inclusion in this meta-analysis based on the following criteria: (a) Population: Studies involving participants from the general population and criminal offenders were included. (b) Study design: Empirical studies that examine corporal punishment and violent crime and report valid effect sizes or other statistical metrics that can be converted into effect sizes were included. (c) Publication Status: Published articles, conference abstracts, and unpublished dissertations or theses were all eligible for inclusion. (d) Language: Studies published in English were included. Studies were excluded if they did not meet the above inclusion criteria or if they were duplicates. Individuals with mental disorders and various types of clinical samples were also excluded. Two independent authors screened the retrieved studies for eligibility, and any discrepancies were resolved through discussion or consultation with a third author (Buscemi et al., 2006).

### 2.1 Data extraction and coding

The coding procedures for this meta-analysis involved the selection of relevant variables and the extraction of data from the included studies. Two independent coders were responsible for the coding process, and discrepancies were resolved through discussion and consensus (Castillo-Montoya, 2016). The details of coding are as follows: (a) Study characteristics: Information was extracted regarding each study’s authors, publication year, and research design. (b) Sample characteristics: Data were recorded (Lipsey and Wilson, 2001) related to sample size and demographics such as age, gender, and cultural background. Sample gender was coded as the percentage of females. Sample age was coded categorically based on participants’ age range. Samples with a mean age below 13 years were coded as “child” (ch). Samples with a mean age below 19 years were coded as “adolescent” (ad). Samples with a mean age of 19 years or above were coded as “adult” (al). Sample cultural background was coded into East and West categories based on geographical proximity and cultural commonalities. China and Korea were classified as East (e). The United States, Spain, Germany, Canada, and Poland were classified as West (w). (c) Effect sizes: the Pearsons’ *r* was utilized as the primary effect size metric to assess the strength and direction of relationships between variables. Effect sizes were extracted from studies. If unavailable, they were calculated from reported statistics (e.g., means, standard deviations, sample sizes) using validated methods (Wilson, 2001). (d) Corporal punishment severity: Corporal punishment severity was categorized into three levels based on the injury inflicted (physically and psychologically)- low-level corporal punishment (LCP), medium-level corporal punishment (MCP), and high-level corporal punishment (HCP). LCP incorporated less than one instance of spanking, deprivation of privileges, inductive discipline, and mild forms. MCP involved more than two instances of spanking, power-assertive techniques, penalty tasks, lax or reactive approaches, verbal punishment, caning, and slapping. Finally, HCP included psychological aggression, harsh corporal punishment, and punitive discipline (Larzelere, 2000). (e) VBS type: VBS type was categorized into four categories based on the severity of VBS, from low to high: anti-social behavior, aggressive behavior, violence, and crime. (f) Additional information: Any additional relevant information, such as subgroup analyses, moderators, or follow-up data, was also coded when available (Lipsey and Wilson, 2001).

### 2.2 Quality assessment

The Troyer scale is used to evaluate the quality of studies in the current meta-analysis (Troyer and Younts, 1997). It involves assessing each study based on several criteria: (a) Sample representativeness and heterogeneity: Studies get 2 points for having a representative, heterogeneous sample; 1 point for a moderately representative sample; and 0 points for an unrepresentative sample. (b) Effect size calculation: Studies get 2 points for using appropriate statistical methods to calculate effect sizes; 1 point for using basic methods; and 0 points for not providing effect sizes. (c) Peer-review: Studies published in peer-reviewed journals get 1 point; non-peer-reviewed studies get 0 points. (d) Sample size: Studies with *n* ≥ 100 get 2 points; studies with 50 ≤ *n* < 100 get 1 point; studies with *n* < 50 get 0 points. (e) The points are summed to give an overall quality rating: 7–8 represents high quality; 4–6 represents Medium quality; 0–3 represents Low quality. By assessing indicators like sample characteristics, statistical analysis, peer review, and sample size, the Troyer scale provides a relatively comprehensive measure of study quality for meta-analyses.

### 2.3 Meta-analysis procedure

The coded data were synthesized using meta for packages in R (R Core Team, 2016). Given that some effect sizes are got from the same sample in current data, the three-level meta-analytic methods with random effect size model were used to calculate overall effect sizes and assess heterogeneity among studies, because we hypothesis that there are significant heterogeneity exist. Three-level meta-analysis is a statistical technique employed in meta-analytic research to address the potential issue of non-independence among effect sizes derived from the same sample. To accommodate this non-independence, three-level meta-analysis employs a more sophisticated approach to estimating the overall effect size and its associated uncertainty. Before the formal meta-analysis, each correlation coefficient underwent Fisher’s z-transformation (Gleser and Olkin, 2009), due to the non-normal distribution of correlation coefficients, unless the population correlation coefficient equates to zero. Consequently, this weighting scheme led to larger-scale studies exerting a more substantial influence during the pooling process. Additionally, we conducted an exploration of potential outliers using studentized residuals and executed leave-one-out sensitivity analyses (Viechtbauer and Cheung, 2010).

To explore potential factors that might influence the relationship, we conducted several meta-regressions with categorical variables are dummy codes. To address the issue of Type I errors in analyzing the dummy coded categorical variables, which can arise from multiple comparisons, we applied Bonferroni correction to adjust the probability values (Jafari and Ansari-Pour, 2019). Publication bias is a significant concern in meta-analytic research. It can distort effect size estimates and lead to incorrect conclusions (Begg and Mazumdar, 1994; Egger et al., 1997). To tackle this issue, we examined publication bias using various diagnostic methods as follows: we generated funnel plots for effect sizes, displaying effect size estimates against their standard errors. A symmetric funnel plot suggests a lower likelihood of publication bias, while asymmetry may indicate the presence of bias (Sterne et al., 2011). We also used the recommended adapted Egger’s regression test to Diagnostics the possible of publication bias (Rodgers and Pustejovsky, 2020). This method is proved to be more suitable to deal with meta-analytic data with multi-level (Ditzer et al., 2023). the tim-fill method and Rosenthal’s fail-safe N method are also used for detecting publication bias (Rosenthal, 1979; Duval and Tweedie, 2000).

## 3 Results

### 3.1 Inclusion of studies and coding consistency

Our initial search was conducted up to July 2023, and subsequently, we conducted an updated systematic search for any newly published studies up to January 2024. This comprehensive search generated a total of 4,833 results. After a meticulous review, we eliminated 1,660 duplicate records and excluded 2,573 studies that did not align with our inclusion criteria.

Finally, we proceeded to assess 600 articles through a thorough examination of their full texts. Unfortunately, 565 of these studies did not provide the essential effect size data required for our analysis. Ultimately, we included a total of 35 pertinent studies, encompassing 144 effect sizes involved 159,213 participants.

**Figure 1:**
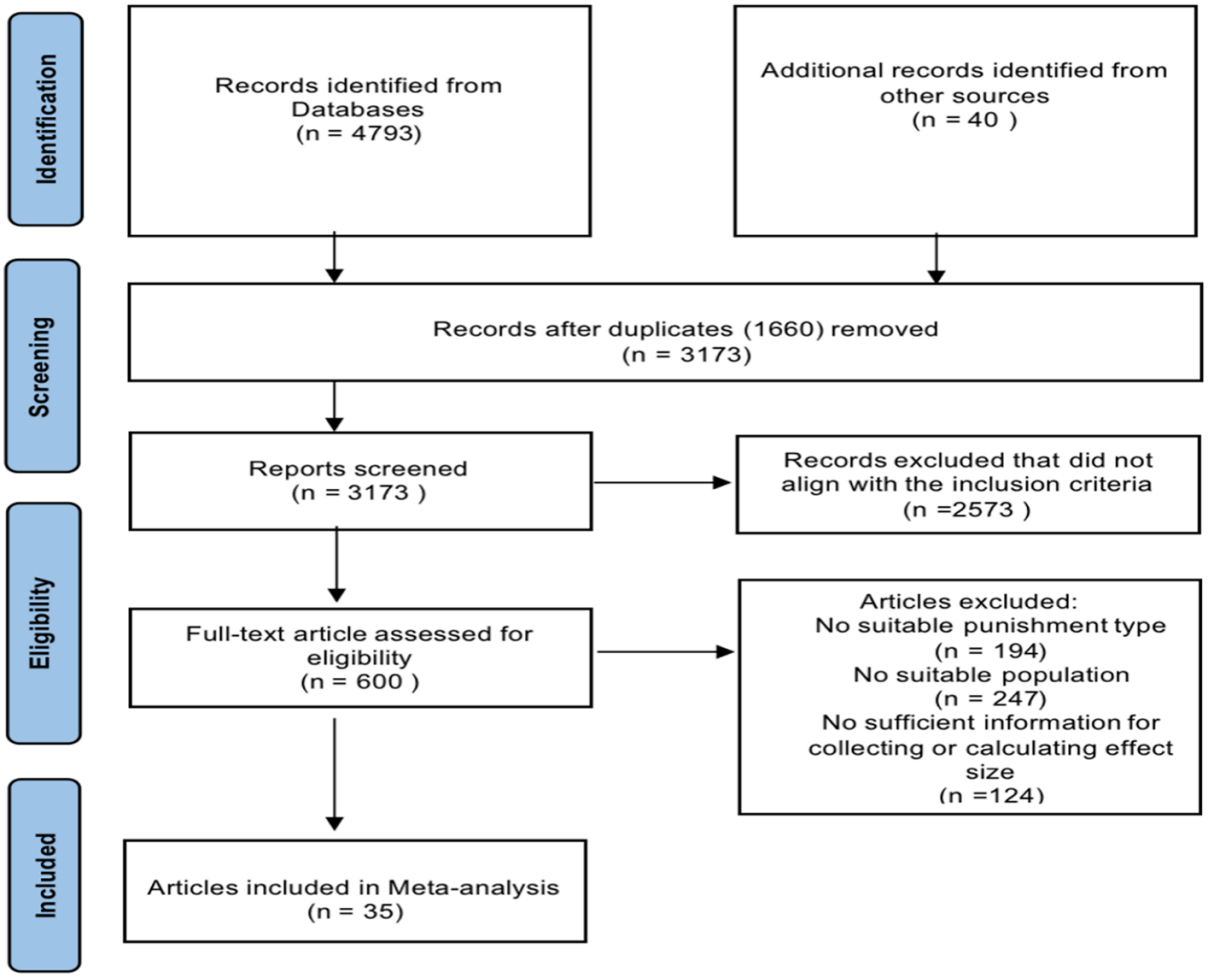
PRISMA flow diagram for new systematic review.

For transparency, we have provided a visual representation of the document inclusion process in Figure 1. It was a high level of agreement, reaching 89%, between the two coders in the selection of relevant literature. To ensure the reliability of our coding process, we utilized Kappa statistic and the Intraclass Correlations Coefficient (ICC). The consistency and agreement in our coding were robust, with values ranging from 0.84 (Kappa) to 0.96. Any discrepancies in coding were promptly resolved through collaborative consensus discussions. Comprehensive information regarding the studies that were included in our analysis can be found in Table 1.

**Table 1:** Comprehensive information of included studies.

Author(s)	Pub_year	*K*	*n*	Gender	Age	State	VBS_type	Pun_lev
Boutwell	2011	1	10,700	49	4	USA	Anti-social	MCP
Taylor et al.	2010	5	2,461	48.1	5	USA	Aggression	MCP
Temple and Scott	2017	2	758	61	20	USA	Violence	MCP
Zottis et al.	2013	18	247	53	12.5	Brazil	Violence	MCP
Hecker et al.	2013	4	409	48	10.49	Africa	Aggression	LCP
Pagani et al.	2004	2	1,175	52	15.7	Canada	Aggression	HCP
Morrison and Gibson	2015	3	704	0	11	USA	Anti-social	HCP
Proulx et al.	2018	2	9,376	70	21	China	Crime	LCP
Liu	2015	8	2,707	0.45	13.5	China	Aggression	LCP
Ma et al.	2018	4	2,472		4	USA	Aggression	MCP
Beckmann	2019	2	7,423	52.3	14.9	Germany	Crime	MCP
SimonsandSutton	2020	3	318	0	23.75	USA	Crime	MCP
Li et al.	2021	1	3,180	47.48	14.93	China	Aggression	MCP
JoynerandBeaver	2022	2	5,827	50	7	USA	Crime	MCP
Straus et al.	1997	20	380	0	4	USA	Anti-social	MCP
Liu et al.	2022	2	2,075	100	13.95	China	Violence	LCP
Ma et al.	2022	1	2,180	49	6.5	Korea	Aggression	LCP
Chen and Pan	2021	1	433	50.6	9	China	Violence	MCP
Lozano and Contreras	2021	2	1,543	49.8	19.9	Spain	Violence	HCP
Gunnoe and Mariner	1997	3	1,112	100	7.5	USA	Aggression	LCP
Sears	1960	4	160	100	12	USA	Aggression	MCP
Slade and Wissow	2003	1	1,966	50	17.5	USA	Aggression	MCP
Lee et al.	2014	4	1,298		3	USA	Aggression	MCP
Joana et al.	2018	4	896	58.8	14.9	Spain	Aggression	MCP
Zulauf et al.	2017	4	240	49.2	3.5	USA	Aggression	MCP
Lee et al.	2013	6	3,279	48	4.7	USA	Aggression	MCP
Berlin et al.	2009	12	2,573	49	2	USA	Aggression	MCP
Ortiz et al.	2015	2	2,060	47.9	14.34	Spain	Violence	MCP
Wang et al.	2016	1	1,971	49.3	12	China	Aggression	MCP
Avinun et al.	2017	2	875	50	6.5	Israel	Aggression	MCP
Liu et al.	2021	1	1,635	54.6	14.24	China	Aggression	MCP
Cresent	2005	4	286	50	5	China	Aggression	MCP
Zhu et al.	2017	9	342	48.8	12.4	China	Aggression	MCP
Lansford et al.	2014	3	85,999	50	8		Aggression	LCP
Monika et al.	2016	1	153	51.2	21.45	Polish	Aggression	MCP

pub_year represents the publication year; k represents the effect size number in one study. n represents the sample size; Gender represents the percentage of females in the total sample; Age represents the average age of the participants; Pun_lev represents the severity of corporal punishment; LCP represents low-level corporal punishment; MCP represents middle-level corporal punishment; HCP represents high-level corporal punishment. VBS_type represents the type of VBS, the range from mild to severe is as follows: anti-social behavior, aggression, violence, and crime.

### 3.2 Overall effect size

The primary effects analysis incorporated 35 pertinent studies, encompassing 144 effect sizes. Sensitivity analysis (with studentized residuals > 2.5 and Cook’s *d* value > 0.4) found no outlines. To account for the anticipated presence of moderators that could contribute to effect size heterogeneity, we employed a random effects model for the meta-analysis. The combined effect size of corpral punishment and VBS was calculated to be *r* = 0.238, with a 95% confidence interval of [0.176, 0.300]. This substantial effect size is indicative of small but significant positive relationship between corpral punishment and VBS as recommended by Lipsey and Wilson’s criterion for defining a high correlation (Lipsey and Wilson, 2001). In terms of the distribution of variance, approximately 9.49%of the variance is attributed to within-study factors (*I*^2^_level2_), while 89.6% is attributed to between-study factors (*I*^2^_level3_). This result provides robust support for Hypothesis.

### 3.3 Result of publication bias

**Figure 2:**
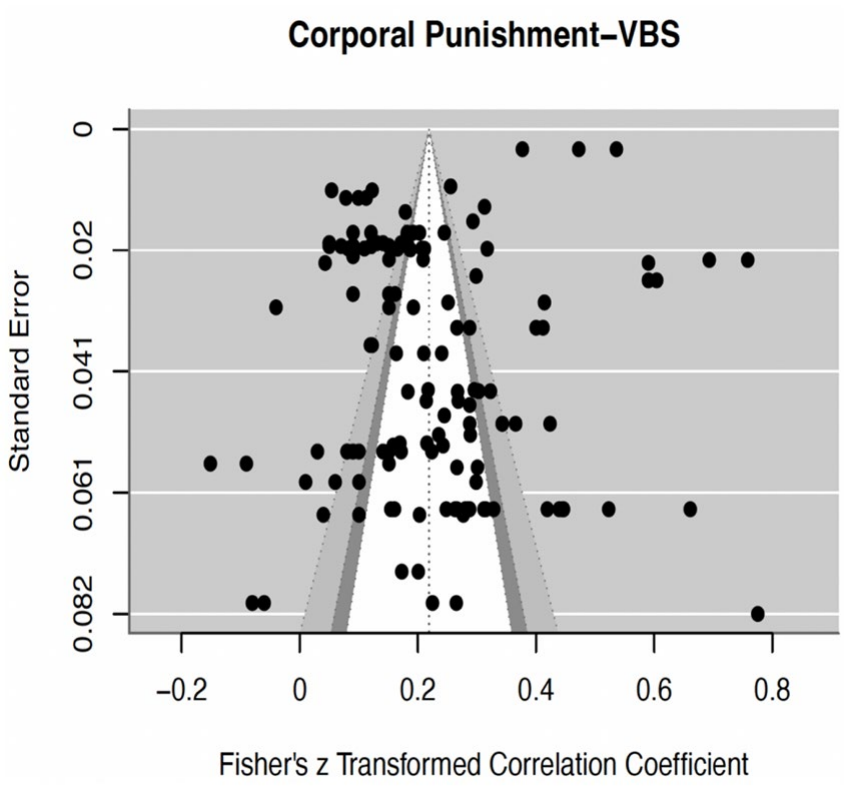
The contour-enhanced funnel plot. The y-axis displays the standard error, while Fisher’s z is plotted on the x-axis. The observed effect sizes are represented by black dots. The overall mean effect is depicted by a middle dashed line. The contour lines, progressing from inner to outer, delineate the 90%, 95%, 99% pseudo confidence interval regions.

In our assessment for the potential presence of publication bias, we employed a multifaceted approach. First and foremost, we scrutinized a funnel diagram (Figure 2) as an integral component of our analysis. Additionally, Due to the nested structure of the data, the traditional Egger’s test for publication bias is not applicable. Therefore, we employed the latest MAML Egger’s test, which uses the standard error of the effect size as a function (Rodgers and Pustejovsky, 2020). If the regression coefficient of the effect size on the standard error variation is significant, it indicates the presence of significant publication bias. The results of yielded a *b* value of −0.4126 (*p* = 0.500) which shown little evidence for publication bias. Thirdly, we applied the trim-fill method to estimate the number of potentially missing studies on the opposite side of the funnel plot (the number is 0) (Duval and Tweedie, 2000). we also conducted an analysis using Rosenthal’s fail-safe N (safety number) (Rosenthal, 1979). The result revealed a Fail-safe N value of 1,757,217, which is greater than 5*k + 10, where “k” represents the number of observed studies. All of these methods consistently support our conclusion that there is little evidence of substantial publication bias impacting our study results.

### 3.4 Results of main effect

The results were obtained from the random effect models of three-level meta-analyses. For the overall model, the result showed a positive correlation (*r* = 0.238; 95% CI = [0.176, 0.300]), exhibiting a small effect size (Cohen, 1992). As for the LCP model, the result showed a positive relationship (*r* = 0.220; 95% CI = [0.089, 0.350]), exhibiting a small to medium effect size. As for the MCP model, a positive relationship (*r* = 0.225; 95% CI = [0.162, 0.288]) was found, exhibiting a small to medium effect size. Furthermore, the pooled effect size of the HCP model showed a positive relationship, but the association is not significant (*r* = 0.318; 95% CI = [−0.128, 0.764]). Table 2 depicts the results of the main effect analyses.

**Table 2:** Pooled effect sizes of corporal punishment-vbs association.

Model types	*k*	#ES	*r*	95% CI	*I* ^2^_level 2_	*I* ^2^_level 3_	σ^2^_level 2_	σ^2^_level 3_	*Q*
Overall model	35	144	0.238^***^	[0.176, 0.300]	9.49%	89.6%	0.032	0.003	14373.910^***^
LCP model	5	12	0.220^***^	[0.089, 0.350]	17.98%	81.77%	0.018	0.004	2581.875^***^
MCP model	123	31	0.225^***^	[0.162, 0.288]	7.80%	90.28%	0.030	0.003	3127.145^***^
HCP model	9	3	0.318	[−0.128, 0.764]	17.28%	81.00%	0.010	0.021	191.669^***^

k: Number of studies; #ES: Number of effect sizes; r: Mean effect size after correcting for measurement error; 95%CI: 95% confidence interval of r; *I*^2^_level 2_: Percentage of variance distributed at the within-study level; *I*^2^_level 3_: Percentage of variance distributed at the between-study level; σ^2^_level 2_: Variance of effect sizes extracted from the same study; σ^2^_level 3_: Variance of effect sizes between studies; Q: Q-statistic magnitude used to assess the heterogeneity of effect. *p < 0.05. **p < 0.01. ***p < 0.001.

### 3.5 Result of meta-regression

In order to thoroughly investigate potential moderating factors influencing our research, such as publication year, age, gender, culture VBS type and punishment severity, we carried out comprehensive meta-regression analyses. The outcomes of these analyses are summarized as follows (see Table 2): (a) Publication year (*b* = 0.005, *p* = 0.092) did not demonstrate a statistically significant moderating influence on the relationship under investigation. (b) With adulthood as the reference category, developmental stage exhibited non-significant moderating effects for both children (*b* = −0.60, *p* = 0.416) and adolescents (*b* = 0.082, *p* = 0.404) on the relationship. (c) Gender (*b* = 0.002, *p* = 0.067) did not yield a statistically significant moderating effect. (d) Culture (*b* = 0.029, *p* = 0.679) also failed to exert a statistically significant moderating impact. (e)With aggression as the reference category, VBS type exhibited non-significant moderating effects for anti-social behavior (*b* = 0.095, *p* = 0.166), Violence (*b* = 0.072, *p* = 0.471) and Criminal behavior (*b* = −0.133, *p* = 0.182) on the relationship. (f) With low punishment level as the reference category, punishment level exhibited a significant moderating effect for both medium punishment level (*b* = 0.084, *p* = 0.010) and high punishment level (*b* = 0.134, *p* = 0.004) on the relationship. The analysis revealed a noteworthy finding, indicating that punishment severity indeed exerts a statistically significant moderation effect on the relationship, signifying its importance in our study. We also performed subgroup analyses to uncover the specific effect sizes associated with each category of moderators (refer to Table 3).

**Table 3:** Results of moderators for the effect sizes.

Moderators	*B*	SE	*p*
Publication year	0.005	0.003	0.092
Gender	0.002	0.001	0.067
**Development stage (adult as reference category)**			
Children	−0.060	0.074	0.416
Adolescent	0.082	0.098	0.404
**Culture (East culture as reference category)**			
West culture	0.029	0.071	0.679
**Punishment level (Low punishment level as reference category)**			
Medium punishment level	0.084**	0.032	0.010
High punishment level	0.134**	0.046	0.004
**VBS type(aggression as reference category)**			
Anti-social behavior	0.095	0.068	0.166
Violence	0.072	0.099	0.471
Criminal behavior	−0.133	0.099	0.183

Gender was coded as the proportion of female participants in the study sample. Development stage was categorized based on subjects’ age, grouped as child (age < 13), adolescent (age < 19), or adult (age ≥ 19). Culture was classified based on the country in which the study was conducted; countries were grouped into East and West categories based on cultural commonalities. Punishment level was categorized as low, medium, or high based on the frequency of punishment enacted and degree of pain inflicted, both physically and psychologically. VBS type was categorized as anti-social behavior, aggression, violence or crime according to the severity of the VBS.

## 4 Discussion

This study employed a three-level meta-analysis system to delve into the relationship between corporal punishment and the Violent Behavior Spectrum (VBS). The findings revealed a statistically significant but modest effect size between the two variables. These results are consistent with previous empirical studies and meta-analyses, providing further support for social learning theory as a relevant framework. Moreover, the analysis of moderating effects shed light on the significant impact of the intensity of corporal punishment on the relationship between corporal punishment and VBS. This finding aligns with previous studies and offers valuable insights into the nuanced boundary conditions of the relationship between corporal punishment and VBS (Ferguson, 2013; Patrick and Drislane, 2015). From a practical perspective, these research findings carry substantial implications for guiding future interventions and developing programs aimed at preventing and addressing the VBS.

### 4.1 Corporal punishment and VBS

The current findings revealed a positive correlation between corporal punishment and VBS. The findings align with prior research (Gershoff, 2002). This consistency across studies underscores the robustness of this association and provides a solid foundation for our exploration of the intricate mechanisms that underlie this relationship. As we delve deeper into the theoretical underpinnings, it becomes evident that social learning theory offers invaluable insights into explaining the observed correlation. This theoretical framework, firmly grounded in Bandura’s influential work (Bandura and Jeffrey, 1973), emphasizes the role of observational learning in shaping behavior. In the context of corporal punishment, it proposes that when children are exposed to adults employing violence as a disciplinary tactic, they become susceptible to modeling this behavior. The process is akin to a form of social mimicry, where children internalize the observed violent actions and subsequently replicate them in their own interpersonal interactions. Therefore, the positive correlation between corporal punishment and VBS can be attributed to this intricate social learning process. Children, as per this theory, may perceive violence as a legitimate and effective means of problem-solving or conflict resolution because they have witnessed its application by authority figures, such as parents or caregivers. This modeling effect can lead to the adoption of violent behaviors as a learned response to challenging situations, thereby strengthening the link between corporal punishment and the manifestation of VBS. Simultaneously, this study deepens our understanding of the theory of socio-moral development. Rewards and punishments can be used to uphold societal moral standards. When the purpose of punishment is to aid children in learning moral norms and the severity is relatively low, it may not increase aggressive behavior in children. However, highly severe and frequent punishment can contribute to subsequent aggression (Baker and Liu, 2021; Geraci et al., 2023).

### 4.2 Punishment severity as a moderator

In our study, we have uncovered pivotal moderating influences that shed light on the relationship between corporal punishment and the Violent Behavior Spectrum (VBS). These insights provide invaluable guidance for understanding the intricate dynamics at play.

Control theories posit that corporal punishment, when judiciously administered without excessive severity, can serve as a tool to reinforce self-regulation and potentially deter future antisocial behavior (Gottfredson and Hirschi, 1990). This perspective hinges on a crucial distinction between the consequences of mild versus severe punishment, aligning with contrasting theoretical models—one emphasizing learning and the other focusing on control. Our findings align with this theoretical framework and resonate with extensive empirical evidence. Notably, Gershoff’s meta-analysis established a clear dose–response relationship, linking more severe and frequent corporal punishment to higher levels of child aggression (Gershoff, 2002). Longitudinal studies have similarly highlighted that experiences of physical abuse during childhood correlate with subsequent involvement in violent crimes, whereas mild spanking does not share this association (Ferguson, 2013). This divergence in outcomes can be attributed to the fact that severe punishment may trigger hostile attribution and heightened rejection sensitivity, whereas milder disciplinary measures offer an avenue for learning without causing trauma (Xu et al., 2023).

### 4.3 Limitations and future directions

Despite providing valuable insights, this meta-analysis has limitations that present intriguing future research directions: (a) Included studies exhibited methodological and demographic homogeneity. Examining more diverse studies conducted in varying cultural contexts and age groups would be beneficial; (b) The correlational nature of included studies precludes determining causality definitively. Longitudinal designs could offer deeper understanding of the temporal relationship; (c) Measurement heterogeneity may have introduced bias. Standardized, consistent tools could enhance comparability; (d) Context-specific corporal punishment approach impacts on violent tendencies may be a valuable research avenue; (e) Previous research suggests that children’s understanding of corporal punishment may moderate the relationship between corporal punishment and aggressive behavior. However, quantifying children’s perceptions regarding corporal punishment has posed challenges in past work. Therefore, we did not encode children’s understanding as a moderating variable in our study. Going forward, developing quantifiable measures to assess how individuals perceive and comprehend corporal punishment could prove informative. Incorporating such metrics as moderators may further elucidate the nuances of how corporal punishment potentially impacts aggressive behavior; (f) The relatively few included studies led to insufficient statistical power for some subgroup analyses. Understanding when and where certain disciplinary strategies are more or less effective is essential for practical implications. Further research should also evaluate interventions aimed at reducing corporal punishment and associated outcomes. This would provide actionable insights for policymakers and practitioners.

## 5 Conclusion

In this meta-analysis, we uncovered a mild yet significant correlation between corporal punishment and subsequent violent tendencies, moderated by punishment severity, aligning with theories on conflicting learning and control mechanisms. While more research is warranted, these insights underscore measured, context-aware disciplinary approaches’ importance as we strive to foster safer environments for children and adolescents.

## Data availability statement

The original contributions presented in the study are included in the article/supplementary material, further inquiries can be directed to the corresponding author.

## Author contributions

QP: Conceptualization, Supervision, Validation, Writing – original draft, Writing – review & editing. SC: Conceptualization, Data curation, Writing – review & editing. YQ: Data curation, Writing – review & editing.
